# Healthcare system amidst the war in Ukraine

**DOI:** 10.1016/j.amsu.2022.104271

**Published:** 2022-07-31

**Authors:** Anastasiia D. Shkodina, Hitesh Chopra, Inderbir Singh, Shoaib Ahmad, Dmytro I. Boiko

**Affiliations:** Poltava State Medical University, Poltava, Ukraine; Municipal Enterprise “1 City Clinical Hospital of Poltava City Council”, Poltava, Ukraine; Chitkara College of Pharmacy, Chitkara University, Rajpura, 140401, Punjab, India; District Head Quarters Teaching Hospital, Faisalabad, Pakistan; Poltava State Medical University, Poltava, Ukraine

For more than two months now, Russian troops have been destroying cities in Ukraine. On February 24, 2022, Russia unreasonably attacked Ukraine. The bloody conflict in Ukraine has seen several attacks on healthcare institutions, including hospitals and clinics. Mariupol's children's hospital was targeted, while Ukraine accused Russia of bombing a mental facility in Kharkiv [1]. Since the inception of the war in Ukraine, the healthcare system in some regions has been devastated. As per records, a total of 103 assaults have been documented, including 89 attacks on healthcare institutions and about 13 attacks on healthcare transportation.

While progress was achieved before the conflict, this is a severe setback for the country's ambitions to implement health reforms and attain universal health care. Conflicts throughout the world have resulted in attacks on health. At least 97 people have been killed and 74 injured because of 160 assaults on health care since January 1, 2022, according to the World Health Organization (WHO) [2]. Infrastructural breakdowns in hospitals and clinics will affect healthcare systems, causing hospital employees to depart and leaving hospitals to deal with increasing patient care demands. According to data from the Ministry of Health of Ukraine above 350 subjects of medical infrastructure were destroyed. On April 28, 2022, it was announced that work would begin on the restoration and reconstruction of medical facilities in the liberated territories, which were destroyed as a result of the Russian war against Ukraine. Work is currently underway to establish a plan for the development and rehabilitation of health facilities [3].

The fighting forced hospitals to suspend work on clinical trials; some resumed a few weeks after the start of the war, but not in the same way as before. There are problems with the safety of patients and the research team, the delivery of drugs and laboratory tests, and the difficulties in carrying out the necessary procedures [4].

In addition, hospitals may keep a limited worth of supplies of medications and other consumables on hand. The quick use of medical resources like bandages, needles, and antibiotics during a conflict is regrettable. Patients with long-term illnesses, including those with tuberculosis and people living with HIV, cannot always get the drugs they need promptly. Some of them may find necessary opportunities for treatment abroad [5]. More than 5.4 million refugees have already fled Ukraine, making this the fastest-growing refugee crisis since World War II [6].

Less than two months later, reports of sexual violence began to surface against both women and children, and the elderly. The use of sexual violence against civilians during armed conflict is widespread and has now been recognized as a war crime by the International Criminal Court. At the same time, it is medical problem with far-reaching devastating consequences [7]. Primary health care (PHC) services for women's and children's health are hampered. The long-term effects on mother and child health and death throughout the crisis era are yet unknown.

Respiratory infections like coronavirus might develop in vehicles delivering them to the border. People are in cramped circumstances and vaccination programs are often interrupted by war; therefore, it is apparent that war has a direct impact on infectious disease frequency. Background of COVID-19 may also increase consequences either for mental or for somatic health [8]. Deterioration of water and sanitation services, food insecurity, and major overcrowding in certain regions have been caused by the loss of vital infrastructure, which has been weakened or destroyed. Security issues make it difficult for people to get the treatment they need. Special attention should be paid to the state of mental health during the war against the backdrop of the COVID-19 pandemic due to the increasing frequency of posttraumatic stress disorder, generalized anxiety, depression, somatization, trauma exposure, and functional impairment.

Healthcare is a significant force, especially in the context of military conflict. Not only because it takes care of wounded combatants, but also because it allows the society to continue to function before, during, and after the war. The healthcare system in Ukraine is facing the terrible challenges of war and needs humanitarian aid and the support of the international community.

## Ethical approval

Not applicable.

## Sources of funding

This research received no specific grant from any funding agency in the public, commercial, or not-for-profit sectors.

## Author contribution

Study concept or design - Hitesh Chopra, Dmytro I. Boiko, Shoaib Ahmad.

Data collection and interpretation - Anastasiia D. Shkodina, Inderbir Singh.

Writing the paper - Anastasiia D. Shkodina, Hitesh Chopra, Inderbir Singh, Shoaib Ahmad, Dmytro I. Boiko.

## Consent

Not applicable.

## Trial registry number

None.

## Guarantor

None.

## Declaration of competing interest

Authors declare no conflicts of interest.
